# Relation of clinical context to accuracy of simulator-based blood pressure measurement by first-year medical students

**DOI:** 10.5116/ijme.5c0f.935c

**Published:** 2018-12-21

**Authors:** Yuka Yamazaki, Iku Hiyamizu, Kyoko Joyner, Yukie Abe

**Affiliations:** 1 Department of Medical Education, Tokyo Medical University, Nishi Shinjuku, Shinjuku-ku, Tokyo, Japan; 2 Simulation Center, Tokyo Medical University Hospital, Nishi Shinjuku, Shinjuku-ku, Tokyo, Japan

**Keywords:** Blood pressure, assessment, simulation, medical students, clinical skills

## Abstract

**Objectives:**

To explore the association between clinical contexts and accuracy of manikin blood pressure readings by first-year medical students after first Simulation-Based-Education training.

**Methods:**

This cross-sectional study, in controlled simulation settings, was comprised of 121 first-year medical student participants after their first Simulation-Based-Education training. Divided into three groups (n = 39, 42 and 40), participants measured blood pressure on three simulator arms assigned different clinical contexts: healthy young male, young female with hypotension, and elderly male with hypertension and diabetes. Each group performed the same protocol on three different days. A Chi-squared test was performed for between-day and between-case differences of correct answers, and one-way ANOVA with Bonferroni post-hoc comparisons was performed for manikin-settings deviation (reported Systolic Blood Pressure (SBP) - set SBP) among cases.

**Results:**

The proportion of correct answers of on Day Two was significantly lower than on the other two days (χ^2^_(2, N = 285) _= 0.34, p = .84), but roughly comparable among cases (χ^2^_(2, N = 285)_ = 24.07, p < .001). The mean of the differences of (SBPreported - SBPset) of Case Two (M = -6.68, SD = 8.91) was significantly lower than Case One (M = -3.07, SD = 9.11) and Three (M = -1.63, SD = 7.76) (F _(2, 274) _= 8.68, p < .001).

**Conclusions:**

Although no statistical associations were found between clinical contexts and student performance in blood pressure measurement, student familiarity with diseases may be associated with performance in taking blood pressure. Day Two performance underscores the need to promote student confidence in diagnostic skills.

## Introduction

Simulation-Based Education (SBE) is an effective teaching strategy to facilitate learning, improve clinical knowledge, provide controlled and safe practice opportunities, and aid in the development of strong clinical skills.^1^^,^^2^ Moreover, Simulation-Based Education may improve student learning and skill acquisition by bridging the gap between theory and practice.^3^

In the early 1960s, the US began development of simulators that could mimic certain physiological functions of a living human body and utilized them to improve the practice of clinical procedures.^4^ Thus far, use of Simulation-Based Education for the practice of clinical technique has a limited history in Japan.^5^ For instance, around 2005, the first simulation center for anesthesia was developed.^4^ Although simulators hold promise as a popular and cost-effective training technique, scientific evidence for the effectiveness of Simulation-Based Education use may be compromised by methodological challenges, such as inappropriate research design, small sample size, or misuse of statistical tests.^6^ Therefore, accumulating evidence that can demonstrate whether Simulation-Based Education is a more efficacious and efficient strategy than conventional didactic classroom instruction is crucial.^7^

This article focuses on the use of Simulation-Based Education for Blood Pressure (BP) Measurement by medical students. Hypertension is a global public health issue and contributes to the burden of heart disease, stroke, and kidney failure, often resulting in premature death and disability.^8^ In particular, the Japanese Ministry of Health and Welfare has made a concerted effort to reduce hypertension and stroke since 1960.^9^ As a result, the screening of hypertension, with subsequent diagnosis and management if necessary, is crucial.^10^ Although the measurement of blood pressure is one of the most common medical procedures,^10^ mastery of auscultatory blood pressure measurement can be challenging for young medical personnel.^11^ Currently, Simulation-Based Education is regarded as an effective strategy for learning the skills of blood pressure measurement. For example, a study by Lee^12^ demonstrated that both accuracy and confidence in learning blood pressure measurement by third-year pharmacy students were enhanced using simulators. However, published research validating the inclusion of simulation in the development of blood pressure measurement skills, as well as the transfer of those skills to clinical practice, has been limited.^11^ In addition, most research was concentrated in the area of nursing. For example, Lee and colleagues compared student accuracy in the measurement of blood pressure using both live participants and simulator arm.^12^ Gordon and colleagues investigated the effectiveness of simulation-based blood pressure training among preregistration nursing students.^11^ Moreover, Ballard and colleagues explored the effectiveness of Simulation-Based Education as a supplemental measure to improving blood pressure measurement skills of nursing students.^3^

Previous research targeting medical students is limited. Blood pressure measurement is an important clinical skill for medical students that can be assessed in the objective structured clinical examination (OSCE).^13^ In addition, adhering to recommended guidelines for correct blood pressure measurement is a physician’s responsibility in diverse conditions and circumstances.^10^ Although physicians strive to measure blood pressure correctly, sometimes errors in measurement occur due to inadequate cuff size, the omission of palpation, inappropriate cuff deflation, placement of the stethoscope under the cuff, or failure to remove clothing covering the location of the cuff.^10^^,^^14^ Furthermore, blood pressure measurement errors lead to unnecessary treatment, the adverse effects of a drug, or prolonged hypertension.^14^ Early training is crucial.^10^^,^^14^ The overarching issue is that if medical students are not sufficiently familiar with the basic clinical skill of accurate blood pressure measurement in the early stages of their undergraduate medical school training,^10^ then their skills may not be corrected in postgraduate training.^14^

Regarding Simulation-Based Education for blood pressure measurement by medical students, only Leung and Nicholls reported results of a randomized investigation into the effects of clinical context on simulator-based assessment of blood pressure measurement.^15^ They provided clinical contexts with hypertension, and the clinical contexts influenced student performance.^15^ Also, they reported that a cross-over study with a complete census that assigns all students to all contexts might yield more interesting findings than assigning students to different cases.^15^ Therefore, our study sought to overcome the limitations of the Leung and Nicholls study, in which students were divided into groups and assigned to a specific case.^15^

The objective of our study was to investigate whether there was an association between three different clinical contexts on three simulator arms and the accuracy of blood pressure readings by first-year medical students after students completed one Simulation-Based Learning (SBL) module. Unlike the Leung and Nicholls study,^15^ we conducted a complete census and assigned all students to all context cases. In addition, we added a clinical context with hypotension. We hypothesized that there would be no association between clinical contexts and student performance.

## Methods

### Study participants

This cross-sectional survey focused on 121 first-year medical students who had just finished their first Simulation-Based Learning module. These students were divided into three groups: 39 students on Day One, 42 students on Day Two, and 40 students on Day Three. The same protocols were followed all three days, separated by seven-day intervals. No participants took part in the survey for more than one day.

In the Japanese medical, educational system, applicants can take a medical school entrance examination during their final year in high school.^16^ After entry, students spend six years studying liberal arts, basic sciences, clinical medicine, and bedside medicine.^16^ During a medical student’s first year, course content focuses on liberal arts. As a result, most students have not taken any basic sciences curriculum and therefore have little or no medical knowledge. At the survey time in September 2017, participants had not studied basic sciences or clinical medicine in classroom lectures. However, they had learned blood pressure measurement in the four-day clinical-experience-based trainings from April to May in 2017. On the first and second days of training, students learned blood pressure measurement using palpation and auscultation, respectively. Procedurally, students learned the skill through lectures, a textbook, and a video that outlined the steps in performing a blood pressure assessment. After these lessons, the medical students practiced taking their peers’ vital signs in laboratory sessions. On the third day of training, students practiced taking blood pressure on full-size manikin arms with auscultation. On the fourth day of training, they took blood pressure measurements of simulator arms using auscultation, and their skills were then assessed in the Objective Structured Clinical Examination (OSCE). Participants were not informed of the study design but were asked only to follow instructions.

With regard to the ethical considerations for participants, we contacted all first-year students who had attended exactly one practical training to participate in this survey. Students were informed about the procedure of this experiment, that participation was entirely voluntary, and that non-participation would not affect their grades. In addition, students were anonymous to the research team, and submitted the response papers anonymously. This study received ethics approval from the Ethical Review Board of one medical school, Japan.

**Table 1:** Blood pressure measurement participation rates (%) for a cohort of 121 first-year medical students after one simulation training, by clinical context case and day (2017)

Day	Case One	Case Two	Case Three
	Participation rates % (n)		
Day One	82 (32/39)	82 (32/39)	82 (32/39)
Day Two	71 (30/42)	76 (32/42)	76 (32/42)
Day Three	78 (31/40)	80 (32/40)	80 (32/40)

### Data collection

This study was conducted concurrently with completion of the first simulation-based training that the cohort of medical students received in their schooling, and all measurements took place in one practical training setting. Students were divided into twelve groups and reported blood pressures for three manikin arms, which were each assigned a different clinical context. For each of the three days, a different set of four groups among the twelve were assigned to measure the same three clinical contexts. All three manikins were calibrated to a single blood pressure each day, which was different for each of the three days. All participants practiced a blood pressure measurement with peers again before the experiments, and then they were instructed to measure the blood pressures of the three manikins with a sphygmomanometer in response to the leader, who read aloud three clinical contexts in numerical order for each group. As a concrete process of data collection, a paper was set alongside each of the three manikins, which students were to fill out anonymously. Students first circled the case number on the paper; second, they read the clinical context of each simulator arm; third, they took the blood pressure of the manikin arms with auscultation; fourth, they wrote down the measured blood pressure values on the paper; and lastly, they put the papers into boxes according to case numbers. To increase the participation rate, students were not asked to write down their three blood pressure measurement values on the same paper. Therefore, it was not possible to detect whether there was a pattern of correct or incorrect answers for individual students.

All measurements of blood pressure were recorded in mmHg. The manikin arm was calibrated according to instrument instructions before the study to assure accurate readings. Students used the diaphragm of a dual-headed stethoscope for measurements.

### Cases

Three manikin arms were used by students to take blood pressure measurements in this study. In addition, these three manikins were assigned one of the following three clinical contexts. Case One was a healthy 20-year-old male college student with the only pollenosis; Case Two was an 18-year-old female high school student diagnosed with hypotension and presenting with occasional complaints of vertigo, a feeling of floating, and one episode of losing consciousness three times in a morning assembly; and Case Three was an 80-year-old male diagnosed with diabetes and hypertension for 30 years. He had been treated with oral medicines, but this time was admitted to the Department of Endocrinology and Metabolism for glycemic control.

**Table 2:** Proportion of correct answers in a blood pressure measurement practical exam for a cohort of 121 first-year medical students after one simulation training by day and clinical context case (2017)

Variables	Total	Correct	Incorrect	p-value
Relationship by Days				
Participants n (%)	285 (100)	148 (52)	137 (48	
Day Number				
One	96 (100)	55 (57)	41 (43)	< .001
Two	94 (100)	30 (32)	64 (68)	
Three	95 (100)	63 (66)	32 (34)	
Relationship by Cases (Clinical Context)				
Participants n (%)	285 (100)	148 (52)	137 (48)	
Case Number				
One	93 (100)	48 (52)	45 (48)	.844
Two	96 (100)	48 (50)	48 (50)	
Three	96 (100)	52 (54)	44 (46)	

For each day, the systolic (SBP) over diastolic (DBP) blood pressures were set to the same values for all three manikins (cases) but were adjusted to different values each day (e.g., Day One was set to 120/70, Day Two was set to 130/70, and Day Three was set to 110/60). Student-reported values of both systolic and diastolic blood pressure falling within ±5 mmHg of the target were considered as correct answers (i.e., permitted error of no more than±5 mmHg for either value).

### Analysis method

Using the chi-square test, we compared the proportion of correct answers by cases and days. In addition, since Leven’s test proved equal variance, one-way Analysis of Variance (ANOVA) with a post hoc Bonferroni test was used to identify the significant differences between the means for (reported SBP - set SBP). A previous study showed that the average differences between pre-specified simulator arm settings and student readings were usually greater for systolic blood pressure measurements than for diastolic blood pressure measurements.^17^ Therefore, we adopted systolic blood pressure as the measure of the difference between reported blood pressure and the blood pressure setting on the manikin arm.

The statistical software package SAS (version 9.4; SAS Institute, Cary, NC, USA) was used for all analyses. Except for

the Bonferroni test, p-values less than .05 were considered statistically significant. Also, regarding the Bonferroni test, p = .05/3=0.017 was considered statistically significant.

## Results

The participation rate on Day One for Case One was 82% [n = 32/39], for Case Two was 82% [n = 32/39], and for Case Three was 82% [n = 32/39]. The participation rate on the Day Two for Case One was 71% [n = 30/42], for Case Two was 76% [n = 32/42], and for Case Three was 76% [n = 32/42]. The participation rate on Day Three for Case One was 78% [n = 31/40], for Case Two was 80% [n = 32/40], and for Case Three was 80% [n = 32/40] (Table 1). Results of χ^2^ tests show that the proportion of correct answers on Day Two was significantly lower than those of other two days (Day One was 57% [n = 55/96], Day Two was 32% [n = 30/94], and Day Three was 66% [n = 63/95] ) (χ^2^_(2, N = 285) _= 24.07, p <.001) (Table 2 – Relationship by Days). Results of χ^2^ tests showed there were no significant differences in the proportions of correct answers among the three cases. The proportion of correct answers for Case One was 52% [48/93], 50% for Case Two [48/96], and 54% for Case Three [52/96]) (χ^2^_(2, N = 285)_ = 0.34, p = 0.84) (Table 2 – Relationship by Cases). More specifically, even though Day Two had roughly half the number of correct answers in the combined contexts compared to Day One and Day Three, the proportion of correct answers for each context was roughly the same for all three days.

**Table 3:** The differences of means of difference of (reported SBP - set SBP) between three clinical context cases in a blood pressure measurement practical exam for a cohort of 121 first-year medical students after one simulation training (2017)

Case One		Case Two		Case Three		p-value^*^
Mean	SD	Mean	SD	Mean	SD	
-3.07	9.11	-6.68	8.91	-1.63	7.76	< .001

^*^P-value for case one and case two is .015; p-value for case two and case three is < .001

Leven’s test showed that three groups of systolic blood pressures had an equal variance (F_(__2, 274) _= 2.40, p = .0929). Therefore, we used a one-way ANOVA test, to compare the differences of means of the three groups. The overall mean of the differences of (reported SBP - set SBP) were -3.07 (SD = 9.11) for Case One, -6.68 (SD = 8.91) for Case Two, and -1.63 (SD = 7.76) for Case Three. The result of the one-way ANOVA test with Bonferroni test shows that the mean of the differences of (reported SBP - set SBP) of Case Two (M = -6.68, SD = 8.91) was significantly lower than those of Case One (M = -3.07, SD = 9.11) and Case Three (M = -1.63, SD = 7.76) (F_(2,274)_ = 8.68, p < .001) (Table 3).

### Sensitivity analysis

The sensitivity analysis is a critical method to assess the impact, effect or influence of key assumptions or variations such as different methods of analysis, definitions of results, protocol deviations, missing data, and outliers on the overall conclusions of a study.^18^ We assumed that data on Day Two might have skewed the proportion of correct answers by case, since the proportion of correct answers for all cases on Day Two was significantly lower than those of other days. Thus, we conducted a sensitivity analysis to ensure robustness in study results. To confirm non-differences for the proportions of correct answers within the three cases, we examined whether the day affected the proportion of correct answers with regard to clinical context or not. After deleting Day Two data, to identify the significant differences of the means of deviation for (reported SBP - set SBP), first Leven’s test was conducted. As a result, the equal variance was not proven (F_(__2,181)_ = 3.28 p=.0398). Therefore, we conducted Welch’s test to analyze the differences of the means of deviation for (reported SBP - set SBP) with the three cases after deleting Day Two data. As a result, the overall mean of the differences of (reported SBP - set SBP) were -1.39 (SD = 3.76) for Case One, -4.35 (SD = 6.58) for Case Two, and -0.09 (SD = 5.55) for Case Three. The result of Welch’s test shows that the mean of the differences of (reported SBP - set SBP) of Case Two (M = -4.35, SD = 6.58) was significantly lower than those of Case One (M = -1.39, SD = 3.76) and Case Three (M = -0.09, SD = 5.55) (F_(__2,117)_ = 7.89, p < .001). As in the principal trial which used the data from all three days, we got the same results, demonstrating that Day Two did not significantly affect the proportion of correct answers over the three weeks for any of the clinical contexts. Therefore, the sensitivity analysis confirms the robustness of the study design, confirming the assumption that including Day Two in the clinical context data does not change the study’s overall results.

## Discussion

The following student errors might have caused the low proportion of correct answers on Day Two. First, a significant number of students recorded the same blood pressure values from Day One, based on a comparison of Days One and Two. Blood pressures were set the same for all cases on the same day but were set to a different value for each of three days. On Day One, the value was 120/70 mmHg. On Day Two, there was evidence that about 19% [n = 18/94] of students also recorded 120 mmHg for the systolic blood pressure, with only 18% [n=17/94] reporting the correct value of 130 mmHg. Therefore, perhaps some students did not trust their own skills, but relied on peer information from the previous session. After Day Two, students may have realized that the blood pressure value might have varied among three days, and were more diligent in reporting actual measurements on Day Three. As a result, 49% of participants reported correct systolic blood pressures and 57% reported correct diastolic blood pressures on Day Three. Based on this result, the importance of asserting educational practices that teach students to trust their own skills instead of relying on secondary peer information is clear. Some students may fear failure in a research environment, just as in regular examinations.^19^ Some students may believe that academic dishonesty is necessary to pass an exam.^20^ However, in health profession education, a breakdown in academic integrity may result in substandard or inappropriate patient care, since the dishonest student may ignore clinical findings, or may not acquire the knowledge base to deliver high-quality care.^20^ To avoid academic dishonesty, professional ethics must be developed through education, which also helps students increase confidence in their own performance.

Second, a possible factor in student performance was the influence of the leader: on Day Two, the leader who gave instructions to students was different from the leader on the other two days. In addition, students were not familiar with this leader, and therefore, might not have followed this leader’s instruction as rigorously. For example, the participation rate on Day Two was lower than that of the other days. According to Sun and colleagues, students sometimes disregard instructions from certain kinds of teachers, with the most unacceptable behaviors inside the classroom being “disrespecting teachers”. or “refusing carry out instructions”.^21^ Students may exhibit rebellious behaviors towards both teachers who are too gentle, or too rigid but not convincing.^21^

Third, a psychological factor may have prevented students from focusing on the task of blood pressure measurement. On Day Two of the blood pressures practice, September 13, 2017, the powerful Typhoon Talim^22^ was with a few days of reaching Japan. Although the rain had not begun, public notice and safety preparations had begun, creating possible student distraction and worry. However, given that students remained engaged in other school tasks, this psychological explanation might be weak.

Fourth, machine error should be considered. With regard to calibration, staff properly calibrated simulators before the experiments on all days and checked whether they could hear the sounds correctly. Machine error is likely negligible for the following reasons. First, preparation of simulators was not problematic. Second, a closer examination showed that simulators, themselves, did not have problems. For example, simulators did not have a problem outputting higher blood pressures, nor did they make other unintended noises. In addition, the approaching Typhoon Talim caused barometric pressure to vary from the other days,^22^ but according to the manufacturing company, barometric pressure should not affect properly-calibrated machine function. Therefore, machine error causing a lower proportion of correct answers is likely negligible.

With only one Simulation-Based Education unit for training, first-year student performance was not inordinately affected by the clinical contexts. Student proportions of correct answers were not overly different among contextual cases. On the other hand, Leung’s study showed that student performance was strongly affected by the lead-in statements, with the proportion of correct answers for a case with hypertension at about 30%, and that of a healthy young adult at 70%.^15^ Although the participants in both studies were first-year medical students with similar characteristics, the results between Leung’s study and ours were significantly different. Potential reasons for the different results could be first, a performance by our cohort was better than the cohort in Leung’s study, perhaps due to a more effective classroom approach, or second, a difference in overall medical knowledge at that point in training between the two groups may have existed.

In Japan, first-year students in the present study were not assumed to have any significant medical knowledge at survey time. Therefore, a typical blood pressure range for hypertension and hypotension might not be generally known. Note that English presents an extra layer of ambiguity about context, using the terminology “hyper” and “hypo”, which do not provide commonly-used cues readily associated with “high” and “low”, respectively. However, in Japanese, the term for hypertension is “高血圧”, three characters that translate literally to “high blood pressure”. The same is true for hypotension, which is “低血圧”, and literally translates to “low blood pressure”. Therefore, the word cues in Japanese, as well as Chinese as in Leung’s study, are more definitive than in English and can improve the likelihood of context associating student measurement.

Regardless of the three clinical contexts, a student might measure the blood pressure of three manikin arms without taking into account the meaning of the clinical contexts, either due to a lack of understanding of the clinical condition or novice inattention. On the other hand, Leung’s study involved students in Hong Kong, who might already have studied basic sciences, and therefore knew what hypertension was.^23^ Because of their medical knowledge, their performance might have been improved by greater familiarity with clinical contexts. As a further research project, we will continue evaluating the skills in blood pressure measurement for this cohort of students. In a future study, we plan to examine in greater detail how medical context associated with student clinical knowledge can influence blood pressure measurement performance as the student’s education advances.

Although the proportion of correct answers in each of the three cases was not available due to the research design, the systolic blood pressure for the hypotension case (Case Two) was significantly lower than the other two cases, as would be expected. This difference might not arise from student instruction in medical school, but personal daily experience. Young people sometimes suffer from syncope because of orthostatic hypotension,^24^ and students might have seen classmates who have lost consciousness. Students may be more familiar with hypotension than hypertension, and therefore more likely to record, intentionally, a lower value of blood pressure for the female high school student than the healthy young male. From this result, it could be established that medical student prejudice from clinical context may be related to their performance, as in Leung’s study.^15^

However, learning correct technical procedures before medical students start clinical training is essential. For example, Gazibara and colleagues reported that medical students tend to focus more on clinical procedures that do not primarily involve blood pressure measurement after clinical training starts.^10^ As a result, a theoretical perspective to accurate blood pressure measurement tends to decrease.^10^ Therefore, learning correct blood pressure measurement in the first year is very crucial. Repeated Simulation-Based Education modules may increase the quality of student skills, and further studies will indicate whether this repetition of training and practice improves student skills of blood pressure measurement or not. A prior study demonstrated a positive effect on the rescuer’s belief in cardiopulmonary resuscitation (CPR) effectiveness, with concomitant improvement in CPR performance and quality with increased repetition of CPR training.^25^

### Limitations

Our study has several limitations. First, the proportion of correct answers on Day Two was lower than that of other days. However, the sensitivity analysis showed the robustness of results when Day Two was included in the statistical analyses. So, data from Day Two did not appear to skew the association of context with blood pressure measurement. Second, results were not generalizable, because participants were all first-year medical students at one medical school. Third, participants with a motivation to study and practice might be more likely to participate in this survey. Therefore, students who do not feel confident taking blood pressure might be less likely to participate in the survey. Fourth, the reported blood pressures were collected by case, and not by individual student, since measurements were anonymous. Therefore, whether a specific participant failed or not could not be discerned. In addition, the reason for failure could not be examined. Fifth, study results do not imply that first-year students properly understand the meaning of clinical contexts. Sixth, this study was a cross-sectional study. Clarifying the effect of clinical context on blood pressure measurement depends on a student’s ability to employ auscultation skills correctly in the first place. However, to confirm the effect of Simulation-Based Education more scientifically, longitudinal studies are needed. Therefore, a follow-up of the first-year cohort of students is essential to evaluate the improvement of skills after repeated simulation-based education. Finally, in this study, every student experienced Simulation-Based Education before skills were measured. Therefore, to more rigorously examine the outcome of Simulation-Based Education preparation, and gauge accumulation of Simulation-Based Education experience, a comparison of student skills gained through simulation versus non-simulation (live subject) training practices is essential. This comparison could be accomplished by conducting a cross-over or comparison study with students at other medical schools that do not currently provide Simulation-Based Education.

## Conclusions

With only one Simulation-Based Education module, over a half of first-year medical students could report correct blood pressure values. Clinical contexts in the three cases were not significantly associated with student performance. However, we found that diseases which students encounter in daily life may have a somewhat stronger association with clinical performance than chronic diseases typically found in older adults. This result implies that before students learn basic sciences and clinical medicine, many are laypersons who can best understand clinical conditions of diseases that they have encountered in their daily life. Therefore, educators should recognize that first-year students may not be aware of the clinical conditions of most chronic diseases, even as educators may think that these conditions are common knowledge. Moreover, due to the lower proportion of correct answers on Day Two, it is possible that students did not have a high level of confidence with their skills, and perhaps relied on peer information. Therefore, education in academic and professional integrity should strive also to help students recognize the importance of having more confidence in their ability and skills. In addition, repeated practice is important for skill improvement, so we will follow up on student skills in blood pressure measurement. In addition, to investigate the more concrete results of training through Simulation-Based Education, a cross-over study would be advisable. In such a study, a comparison of student performance between one cohort of students with Simulation-Based Education and another cohort of students from one or more medical schools that do not utilize Simulation-Based Education training could help demonstrate the effectiveness of simulator training.

### Acknowledgements

We are grateful to all students who participated in the survey, and staff members who supported our survey. Also, we express our appreciation for Laurie Mena’s dedicated work providing English editing and for Huilin Qian’s data entry.

### Conflict of Interest

The authors declare that they have no conflict of interest.
